# Derbid Planthoppers (Hemiptera: Fulgoroidea: Derbidae) Associated with Coconut and Oil Palm in Brazil

**DOI:** 10.1007/s13744-020-00788-5

**Published:** 2020-07-27

**Authors:** M Dollet, E G Fidelis, E Dos Passos, F Da Silva, H P Aberlenc, D A Schurt, B Bahder, L C Diniz, C R Bartlett

**Affiliations:** 1 Cirad, Umr Ipme Cirad/Ird/UM, Campus International, de Baillarguet, TA A/98, 34398 Montpellier Cedex 5, France; 2 Embrapa Cenargen, Brasilia, DF Brasil; 3 Embrapa Cerrados, Brasilia, DF Brasil; 4 Embrapa Tabuleiros Costeiros, Aracaju, SE Brasil; 5 Instituto Federal do Amapá, Macapá, AM Brasil; 6 Cirad, Umr Bgpi, Montpellier, France; 7 Embrapa Roraima, Boa Vista, RR Brasil; 8 Univ of Florida, Fort Lauderdale, FL USA; 9 Univ of Delaware, Newark, DE USA

**Keywords:** Fulgoromorpha, Auchenorrhyncha, Cenchreini, phytoplasmas, lethal yellowing type syndromes, vectors

## Abstract

We present surveys of derbid planthoppers associated with coconut (*Cocos nucifera* L.) and oil palm (*Elaeis guineensis* Jacq.) collected in Northeastern (Sergipe) and North (Pará and Roraima) Brazil. The surveys were intended to contribute to our knowledge of possible vectors of phytoplasmas or other phloem-restricted plant pathogens. Eight derbid taxa were found, two in the subfamily Cedusinae, tribe Cedusini (*Cedusa yipara* Kramer and *C. yowza* Kramer) and six in the subfamily Derbinae, tribe Cenchreini: *Herpis* sp., *Persis pugnax* Stål, *Omolicna anastomosa* (Caldwell), *O. nigripennis* (Caldwell), and two new species in the genus *Agoo* Bahder & Bartlett are described here. Genus-level features between *Omolicna* and *Agoo* are discussed and a key to the species of *Agoo* is provided.

## Introduction

Coconut tree (*Cocos nucifera* L.) is the fourth most important perennial fruit tree in Brazil, with approximately 158,477 ha of cultivated area (IBGE 2018). Eighty-five percent of the plantations cover less than 10 ha and are in the hands of small farmers, the rest concerns big private agro-industrial companies (Fontes & Wanderley 2006, Martins & Jesus Júnior 2014). Over the last 20 years, coconut water has become one of the most important high value-added products of the agroindustry of Brazil (Fontes & Wanderley 2006) and the country is the world’s fourth largest producer of coconuts (FAO 2020).

Given the importance of coconut in Brazil, the arrival of a serious disease such as lethal yellowing (LY) would be catastrophic (Dollet & Talamni 2018). In Jamaica, LY has caused the death of millions of coconuts and it has destroyed 90% of the coconuts of the Atlantic coast of Honduras in less than 10 years (McGrath 2002, Rocca 2013). LY was reported in more than 30 species of palms in Florida (Dollet & Talamani 2018, Sullivan & Harrison 2013), suggesting that the disease may be a broad threat to indigenous palms in Amazonian region. Valuable palms that might be threatened include the açai palm (*Euterpe oleracea* Mart. and *E. precatoria* Mart.), the buriti palm (*Mauritia flexuosa* L. f.), babaçu (*Attalea speciosa* Burret), pupunha (*Bactris gasipaes* Kunth), and others that are important sources of food and other products (Balick 1979, Kahn 1991, Mtiiz-Miret *et al* 1996, Brondizio 2011, Tunçer & Schroeder 2017).

LY is caused by phytoplasmas that are phloem-limited pathogens transmitted by insects that feed exclusively on phloem tissue (McCoy *et al* 1983, Eden Green 1997, Dollet *et al* 2009). Known vectors of phytoplasmas are in the order Hemiptera, suborder Auchenorrhyncha, mostly families Cixiidae and Cicadellidae (subfamily Deltocephalinae). Derbidae and Flatidae also have been suggested as possible vectors (Mpunami *et al* 2000, Clair *et al* 2001, Wilson 2005, Weintraub & Beanland 2006, Philippe *et al* 2007, Philippe *et al* 2009, Lee & Wilson 2010, Rodrigues *et al* 2010, Rajan 2013, Halbert *et al* 2014).

For this reason, surveys for early detection of LY and potential vectors are being made in Brazil. Here, we report Derbidae found during surveys of coconut and oil palm (*Elaeis guineensis* Jacq.) in Northeastern (Sergipe) and North (Pará and Roraima) Brazil. Two of the species found are described as new species and a key to the species of *Agoo* Bahder & Bartlett is provided.

## Material and Methods

### Study areas and survey methods

**Fig 1:**
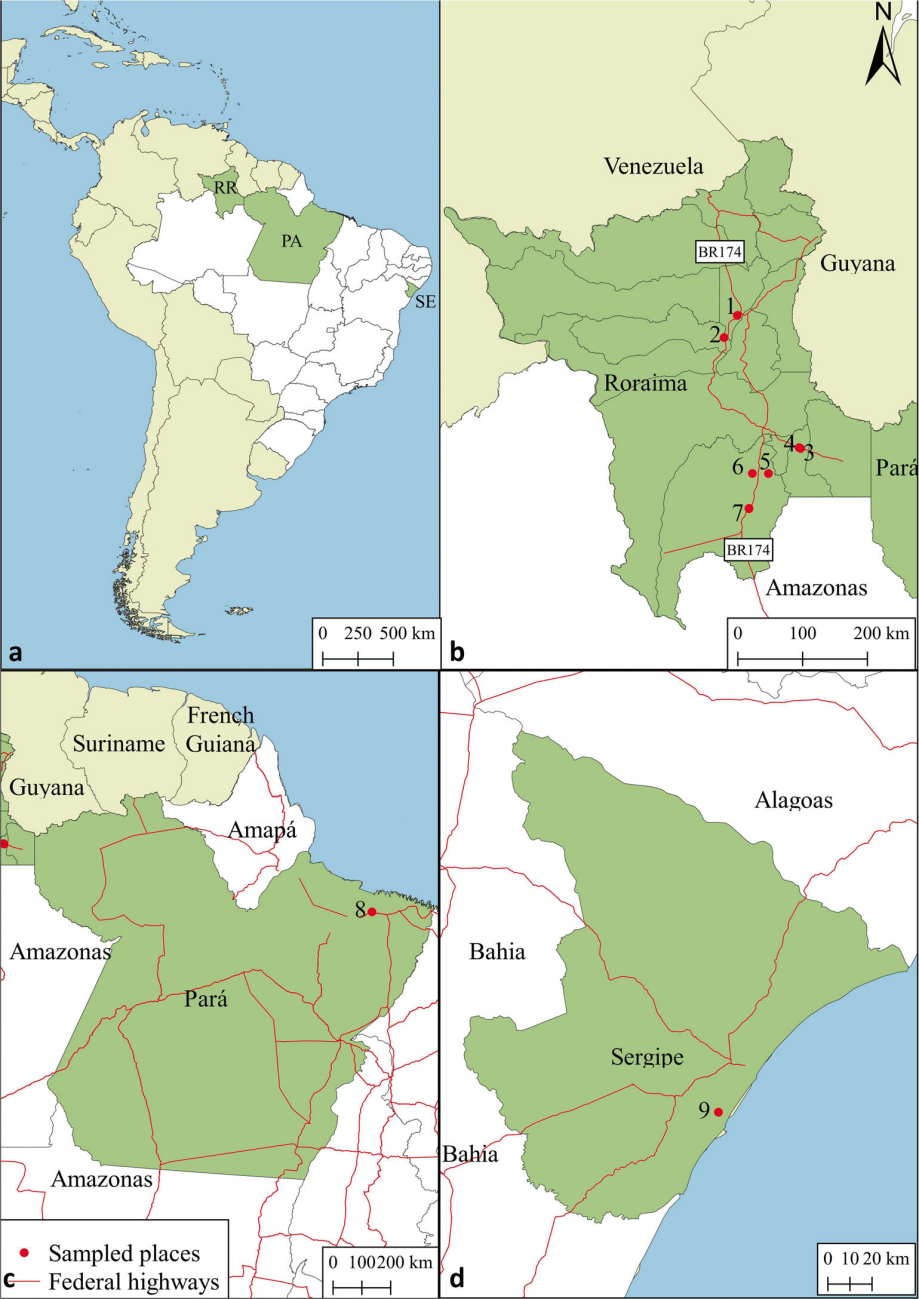
Sample locations for derbid planthoppers: (A) general location of sampling areas (green) in Brazil (white); (B) sampling localities in Roraima; (C) sampling locations in Pará; (D) sampling locations in Sergipe.

Surveys were undertaken in Roraima state, North of Brazil, from July to August 2016 in the following municipalities: Boa Vista, Bonfim, Mucajaí, and Rorainópolis, and in October 2017 in Boa Vista, Cantá, Mucajaí, São João da Baliza, and São Luis (Fig 1). Coconut plantations (*Cocos nucifera* L., Arecaceae) were visited in those municipalities in order to identify plants with LYTS and the presence of potential insect vectors, leafhoppers, and planthoppers. Oil palm plantations (*Elaeis guineensis* Jacq., Arecaceae) were also visited in the south of the State for searching vectors. All plants were observed for symptoms similar to LYTS in leaves of the basal third or at least 20 plants of each plantation. Planthoppers were collected with a mouth-aspirator or caught with a tube and preserved in 70% alcohol.

Derbidae were also collected on the lower leaves of coconuts in the Coconut International Bank for Latin America and the Caribbean (ICG-LAC) located in Itaporanga D’Ajuda, Sergipe State, in Northeastern Brazil. Other derbids were collected using sweeping net and mouth aspirator in a plantation (Brazilian Green Dwarf Jiqui) of Northern Pará state in Santa Izabel do Pará. Insects were collected on the lower leaves of the palms. Information about sampled plantations and geographic coordinates are provided in Table 1 and Fig 1. Derbids were sorted to morphospecies with representative specimens donated to the Museu Paraense Emílio Goeldi, São Bráz, Belém, Pará, Brazil (MPEG), and subsequently loaned to CRB for identification, with representative (non-type) specimens retained at the University of Delaware Reference Collection, Newark, DE (UDCC), by permission of MPEG. Additional study specimens are deposited in Embrapa (Empresa Brasileira de Pesquisa Agropecuária) in Aracaju and Roraima, Brazil.

**Table 1:** Derbidae species associated with coconut and oil palm in Brazil.

Species	Host	Sampled place		
		State/municipality/locality	Geographical coordinates	Number on map (Fig 1)
*Cedusa yipara* Kramer	Oil palm	Roraima, São João da Baliza	0°56′02,3″N, 59°52′39,6″W	3
	Coconut	Roraima, Rorainopolis, Vila Nova Colina	0°35′17,7″N, 60°19′15,8″W	5
	Coconut	Pará, Santa Isabel do Pará, Sococo	01°14.744′S, 048°03′53,3″W	8
*Cedusa yowza* Kramer	Oil palm	Roraima, Rorainopolis, Vila Equador	00°06′12,5″N, 60°35′26,6″W	**7**
*Herpis* sp.	Coconut	Roraima, São João da Baliza	0°56′07,9″N, 59°52′4o,4″W	4
*Persis* (*Persis*) *pugnax* Stål	Coconut	Roraima, Boa Vista	02°47′02,6″N, 60°45′12,5″W	1
	Coconut	Roraima, Mucajaí	02°28′34,9″N, 60°56′11,9″W	2
*Omolicna anastomosa* (Caldwell)	Coconut	Sergipe, Itaporanga D’Ajuda	11°06′14,5″S, 37°11′03,8″W	9
*Omolicna nigripennis* (Caldwell)	Coconut	Roraima, Rorainopolis, Vila Nova Colina	0°35′23,2″N, 60°32′38,6″W	6
	Oil palm	Roraima, Rorainopolis, Vila Equador	00°06′12,5″N, 60°35′26,6″W	7
	Coconut	Sergipe, Itaporanga D’Ajuda	11°06′14,5″S, 37°11′03,8″W	9
*Agoo argutiola* sp. n.	Oil palm	Roraima, Rorainopolis, Nova Colina	00°36′47,85″N, 60°17′40,57″W	3
	Coconut	Roraima, Boa Vista	02°47′02,6″N, 60°45′12,5″W	1
	Coconut	Roraima, São João da Baliza	0°56′54,8″N, 59°53′41,2″W	4
*Agoo spina* sp. n.	Coconut	Sergipe, Itaporanga D’Ajuda	11°06′14,5″S, 37°11′03,8″W	9

### Morphological terminology and specimen techniques

Abdomens were cleared by soaking in 10% KOH solution overnight (or 20% hot KOH for ~ 15 min) for examination. Morphological terminology generally follows that of Bartlett *et al* (2014), except forewing venation following Bourgoin *et al* (2015) and with male terminalia nomenclature modified after Bourgoin (1988) and Bourgoin & Huang (1990). Authorship of the two new species should be attributed to Bahder and Bartlett.

Photographs and measurements were taken using a digital imagery system. Line art was digitally traced from photographs. All measurements are in millimeters (mm). Specimen measurements were taken for descriptive (not statistical) purposes and are from type material.

Label information of holotypes types is quoted, with ‘/’ indicating new line and ‘//’ indicating next label and with supplemental information given in brackets. For other material examined, label data were rewritten to maintain consistency in pattern, beginning with the country, state or province, and more specific locality, followed by the collection date, collector, and lastly, the number, sex of specimens, and specimen depository given in parentheses. Type material and other specimens examined at UDCC were provided with 2D barcode labels and data captured using “Arthropod Easy Data Capture” (Schuh *et al* 2010, Schuh 2012, Arthropod Easy Capture 2013) in the NSF sponsored “Tri-Trophic Thematic Collection Network” (Tri-Trophic TCN, http://tcn.amnh.org/). These data are available via the iDigBio (www.idigbio.org) specimen portal.

### Identification and classification

Derbid specimens were identified to higher taxon following Metcalf (1938) and Fennah (1952) as updated by O’Brien (1982) and Emeljanov (1992, 1996). Species level identifications used available keys and illustrations, and in some cases by comparison with photos of primary type material. The genus *Cedusa* Fowler was identified using Flynn & Kramer (1983) and Kramer (1986); *Omolicna* Fennah by Caldwell (1944), Fennah (1945), Halbert *et al* (2014), and Bahder *et al* (2019); *Herpis* Stål using Metcalf (1938, 1945) and O’Brien (1982, 1987); *Persis* Stål using Metcalf (1938) and Fennah (1945, 1952). The status of *Agoo* Bahder & Bartlett was recently revised by Bahder *et al* (2020a) from a subgenus of *Omolicna* to full genus status. Photos of type material of *Persis pugnax* Stål, *Herpis fuscovittata* Stål, *Phaciocephalus fimbriolata* (Stål), *P. orba* (Stål), and *P. pallidovenosa* (Stål) were obtained from the Swedish Museum of Natural History in Stockholm for comparison with field collected specimens.

## Results

Eight taxa from the family Derbidae were found associated with palms in these surveys (Table 1). Of these, two were in the genus *Cedusa* Fowler (Cedusinae: Cedusini). This genus comprises 148 species in the New World and 32 in Africa, although the latter taxa are probably better placed in *Malenia* Haupt (Fennah 1961, Szwedo 2006). Species of *Cedusa* are often externally very similar and recognition relies almost entirely on features of male terminalia (Flynn & Kramer 1983, Kramer 1986). *Cedusa* has previously been suggested as a possible vector of lethal yellowing phytoplasmas in Jamaica (Brown *et al* 2006). However, no experimental transmission of LY was obtained in Jamaica.

The remaining derbid species were all in the Derbinae, tribe Cenchreini. The extant Cenchreini consists of 22 genera and 186 species, of which 10 genera and 59 species occur in the New World (two genera, as currently defined, occur in both hemispheres) (Bourgoin 2020). These taxa consisted of *Persis punax* Stål, *Herpis* sp. and five species in or near the genus *Omolicna* Fennah.

### Taxa found associated with coconut and oil palm in Brazil

**Fig 2:**
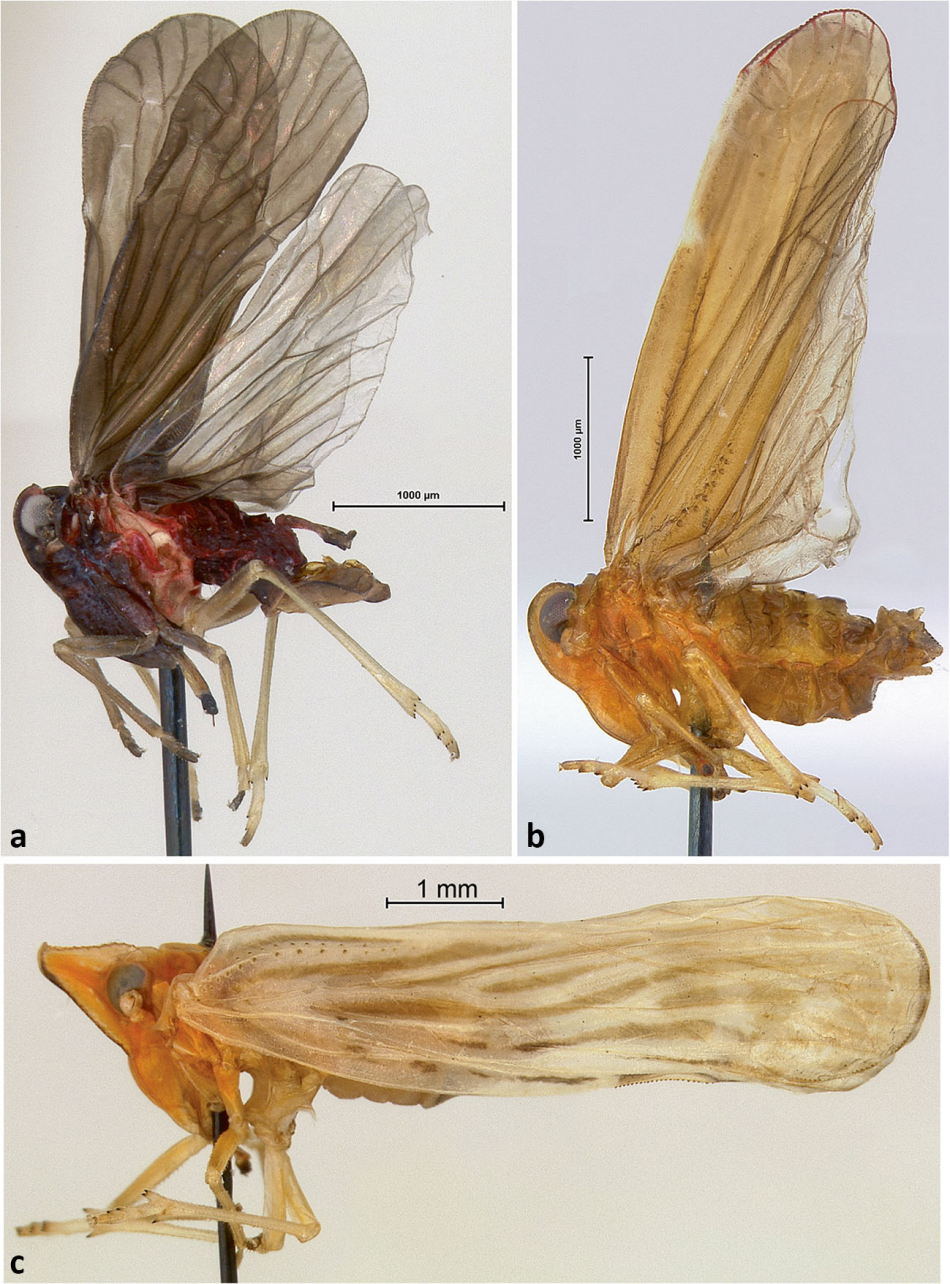
Lateral habitus of derbid planthopper species: (A) *Cedusa yipara*; (B) *Omolicna nigripennis*; (C) *Persis pugnax.*

**Fig 3:**
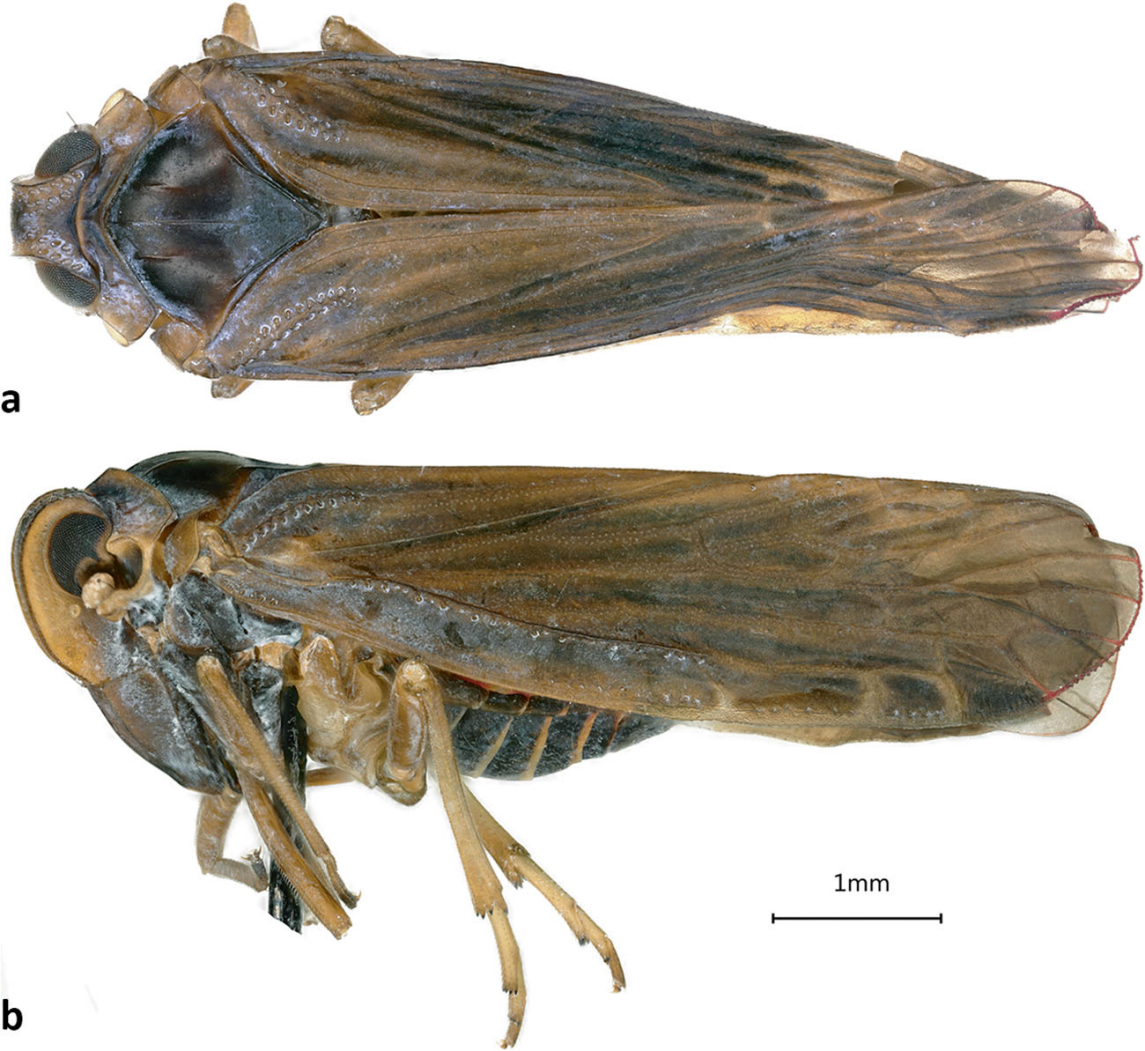
*Herpis* sp.: (A) dorsal habitus; (B) lateral habitus.

**Fig 4:**
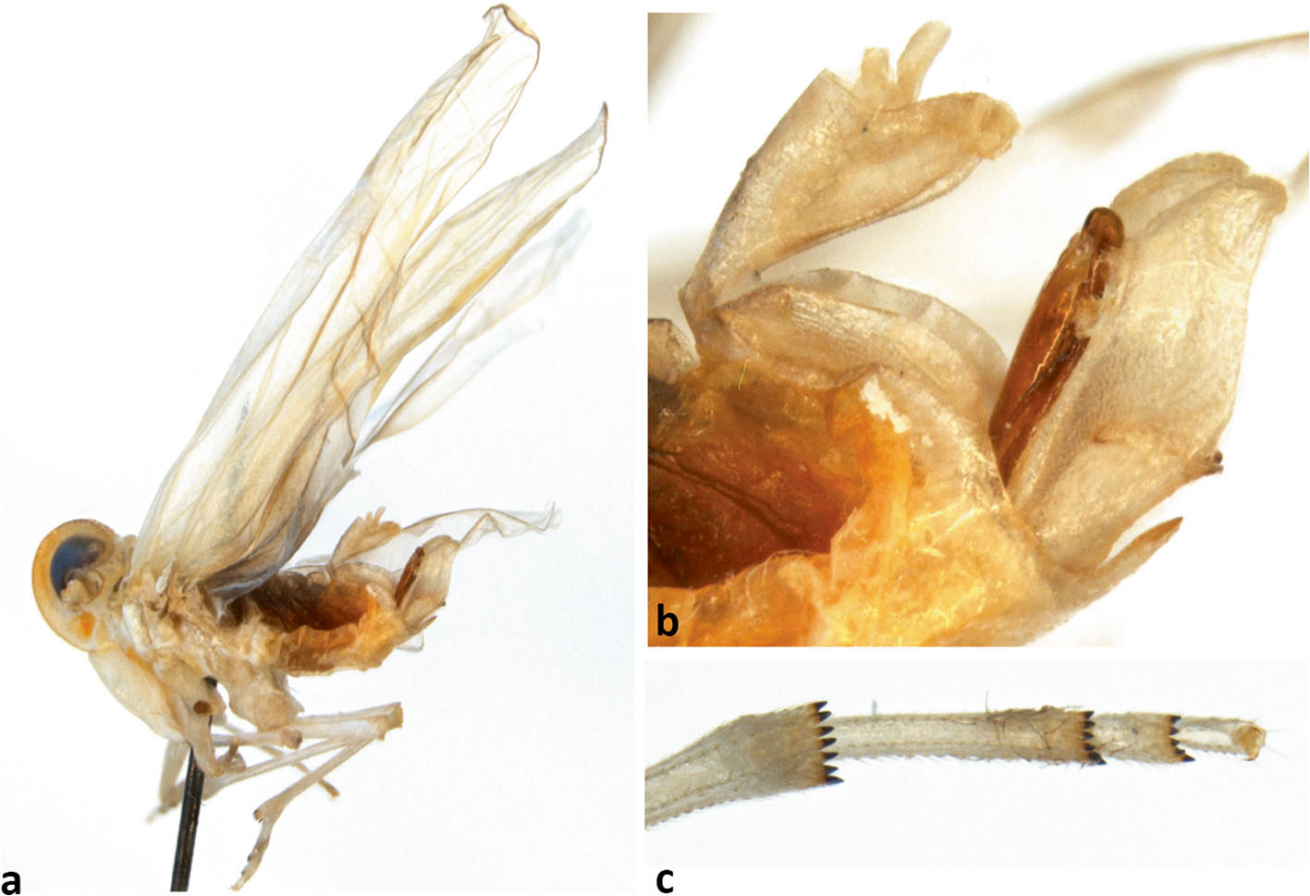
*Agoo argutiola* sp. n. (paratype): (A) lateral view, habitus; (B) terminalia, left lateral view; (C) apex of hind leg, ventral view.

1. ***Cedusa yipara*** **Kramer,** 1986. One specimen (sample SJ1c) (Fig 2A); 20–II–2017; Roraima State, São João da Baliza, 0°56′02.3″N, 59°52′39.6″W, oil palm, Michel Dollet & Elisangela Fidelis leg. One specimen (sample 22936) Rorainopolis, Vila Nova Colina, 01°14.744′S, 048°03.533′W, 3–VIII–2016, coconut, Michel Dollet & Elisangela Fidelis leg.; one specimen Pará, Sococo, Santa Isabel do Pará, 01°14.71030′S, 048°03.533′W, 18–V–2016, coconut, Eliana Passos & Flaviana Silva leg. In addition, there were three females tentatively referred to this species (Sergipe, Itaporanga D’Ajuda, 11°06′14.5″S, 37°11′03.8″W, 22–VII–2015, coconut, Eliana Passos & Flaviana Silva). *Cedusa* Fowler is a difficult genus, and if defined as entirely New World (e.g., by Szwedo 2006, as opposed to the doubtfully distinct *Malenia* Haupt), consists of 148 extant species, most of which are Neotropical. *Cedusa* has been revised by Flynn & Kramer (1983) and Kramer (1986), which collectively treated 147 species in the genus, plus *C. quixoa* Kramer, subsequently moved to *Cedochrusa* Emeljanov (Emeljanov 2008), separated principally by features of the male genitalia. *Cedusa yowza* Kramer (reported below), previously reported from Panama, is (as noted by Kramer 1986) very similar to *Cedusa yipara*, separated from this species by the aedeagal flagellum (endosoma) terminating with semicircular process bearing a subapical fin and apical microteeth (in *C. yowza*, see Kramer 1986, Fig 80), versus flagellum terminating truncately (Kramer 1986, Fig 83). The *C. yipara* specimen from Vila Nova Colina differs in having the apical process on the left side of the aedeagus not distinctly forked and the right paramere not as narrowed. Further investigation is needed to determine the variation found in *C. yipara* and whether there are undescribed species of *Cedusa* on palms in Brazil.
2. ***Cedusa yowza*** **Kramer**. One male, Roraima, Rorainopolis, Vila Equador, 00°06′12.5″N, 60°35′26.6″W, 2–VIII–2016, *Elaeis guineensis* Jacq., Michel Dollet & Elisangela Fidelis leg.
3. ***Herpis*** **sp.** One male (sample SJ4c) (Fig 3), Roraima State in Brazil, São João da Baliza, 0°56′53.3″N, 59°53′42.0″W, coconut, 21–II–2017, Michel Dollet & Elisangela Fidelis leg. The genus *Herpis* Stål consists of the 12 species, three are reported from South America, four are Indomalayan and probably misplaced at the genus level; the balance of species are Mesoamerican. We have not been able to match the single obtained specimens with any of the described species, including any of the three species known from South America. This genus is poorly known from Brazil, with *Herpis fuscovittata* Stål, the only species previously reported from Brazil, and the type species of the genus. We obtained photographs of the type specimen of *Herpis fuscovittata* from the Swedish Museum of Natural History, Stockholm (NHRS-GULI000058748), which showed *H. fuscovittata* to be a much paler species. This specimen does not appear to match the descriptions of either of the other two species reported from South America (viz., *H. chiriquensis* (Fowler), reported from Guyana and Panama, and *H. metcalfi* O’Brien from Guyana (as British Guiana; Muir 1918, Metcalf 1945, O’Brien 1987). The specimen probably represents an undescribed species, but we have not definitively been able to exclude the described Mesoamerican species because of incomplete original descriptions and limited accessibility to type material.
4. ***Persis (Persis) pugnax*** **Stål**. One male (sample 22937) (Fig 2C) from Roraima, Roraima, Boa Vista, 2°47′02.6″N, 60°45′12.5″W, coconut, 26–VII–2016, Michel Dollet & Elisangela Fidelis leg; 2 specimens (1 male, 1 female, 22,940), Mucajaí, Roraima, 2°28′34.9″N, 60°56′11.9″W, coconut, 28–VII–2016, Michel Dollet & Elisangela Fidelis leg. Species identification confirmed by comparison with photographs of two syntype specimens from the Swedish Museum of Natural History, Stockholm (#NHRS-GULI000040282, NHRS-GULI000040281).
5. ***Omolicna anastomosa*** **(Caldwell)**. One male, Sergipe, Itaporanga D’Ajuda, 11°06′14.5″S, 37°11′03.8″W, coconut, 19–V–2016, Eliana Passos & Flaviana Silva, aspirator.
6. ***Omolicna nigripennis*** **(Caldwell)**. One male (sample 22935) (Fig 2B), Roraima, Rorainopolis, Vila Nova Colina, 0°35′23.2″N, 60°32′38.6″W, coconut, 2–VIII–2016, Michel Dollet & Elisangela Fidelis leg; one male (sample 22932), Roraima, Rorainopolis, Vila Ecuador, 00°06′12.5″N, 60°35′26.6″W. Oil palm, Michel Dollet & Elisangela Fidelis leg; Sergipe, Itaporanga D’Ajuda, 11°06′14.5″S, 37°11′03.8″W, coconut, 31–VII–2015, Eliana Passos & Flaviana Silva, sweep net. *Omolicna* specimens identified with Caldwell (1944) using features of male terminalia.
7. ***Agoo argutiola*** **sp. n.** Four males (Fig 4), Roraima, Rorainopolis, Nova Colina, 00°36′48.7″N, 60°17′39.7″W, oil palm, 3-VIII-2016, Michel Dollet & Elisangela Fidelis leg; one male from Roraima, Boa Vista, 2°47′02.6″N, 60°45′12.5″W, coconut, 27–VII–2016, Michel Dollet & Elisangela Fidelis leg; one male (sample SJ4b), Roraima, São João da Baliza, 0°56′07.9″N, 59°52′40.4″W, coconut, 21–III–2017, Michel Dollet & Elisangela Fidelis leg; two females and one male (SJ4d) Roraima, São João da Baliza, 0°56′07.9″N, 59°52′40.4″W, coconut, (20–21)–III–2017 (an additional specimen from this locality appears slightly different and is excluded from the paratype series). This species falls into *Agoo* Bahder & Bartlett (in Bahder *et al* 2019) and is described below. *Agoo* recently changed status from subgenus of *Omolicna* to full genus by Bahder *et al* (2020a).
8. ***Agoo spina*** **sp. n.** One specimen (Fig 8) from Sergipe, Itaporanga D’Ajuda, 11°06′14.5″S, 37°11′03.8″W, coconut, 22–VII–2015, Eliana Passos & Flaviana Silva, sweep net; two specimens same except 19–V–2016, aspirated from coconut. This species similar to *Agoo argutiola* **sp. n.** but differs in details of color and in male terminalia*.*

**Systematics**

**Family Derbidae Spinola, 1839**

**Subfamily Derbinae Spinola, 1839**

**Tribe Cenchreini Muir, 1913**

**Genus** ***Agoo*** **Bahder & Bartlett, 2019**

Type species: *Agoo xavieri* Bahder & Bartlett, 2019

**Amended diagnosis.** The members of *Agoo* are pale forms, with the head in lateral view appearing smoothly rounded from posterior margin of vertex to the frontoclypeal suture. The lateral carinae of the vertex and frons are foliate such that the vertex and frons are concave. The median carina of the vertex and frons appears absent and there is no transverse carina at the fastigium. The frons narrower and paranota more strongly foliate than *Omolicna*. The terminalia are nearly bilaterally symmetrical; the aedeagus is stout with a strongly retrorse flagellum bearing differing, symmetrical arrangements of processes. The ventral lobe of pygofer (ventral view) broad, distally attenuating to rounded apex. Anal tube is stout, elongate, and ventrally sinuate.

**Remarks.** The genus *Agoo* (originally a subgenus of *Omolicna*) is most readily separated from *Omolicna* by the foliate carinae of the head, the profile of the head being strongly rounded, and the more strongly foliate antennal fossae compared with *Omolicna*. The terminalia of *Agoo* are nearly bilaterally symmetrical (more so than *Omolicna*), and the midventral lobe of the opening of the pygofer is elongately rounded, and the anal tube is ventrally sinuate (lacking convexity found in most *Omolicna*). Bahder *et al* (2019) showed a high pairwise distance between *Agoo xavieri* Bahder & Bartlett and five species of *Omolicna*, with a percent nucleotide difference range from 24.4 to 31.1% for CO1 and ~ 10.6% for a 1493 bp region of 18S.

At present, all members of the genus *Agoo* are associated with palms (Arecaceae).

In the key to genera of Cenchreini presented by O’Brien (1982, modified from Fennah 1952), species of *Agoo* key with difficulty because of several ambiguous features (carinae of vertex pustulate; frons narrow, compressed; subantennal fovea present, subcostal cell long, Sc+R fork about level with Cu1 fork and union of claval veins, claval apex in basal half of wing). Depending on interpretation of features, *Agoo* might key to *Cenanges* Fennah, *Neocenchrea* Metcalf, or *Phaciocephalus* Stål. *Cenanges* and *Neocenchrea* can be excluded based on an examination of specific features, especially male genitalia (photos of the type specimen of *Cenanges spectralis* Fennah from The Natural History Museum, London were examined). *Phaciocephalus* appears to be paraphyletic, with 28 species, principally from the Indomalayan region and Oceania except for 3 species (doubtfully included) from Brazil. Photographs of the type specimens of the Brazilian *Phaciocephalus* were obtained from the Swedish Museum of Natural History, Stockholm, to compare with the new species of *Agoo*.

### Preliminary key to separate *Omolicna* and *Agoo* and species of *Agoo*

**Fig 5:**
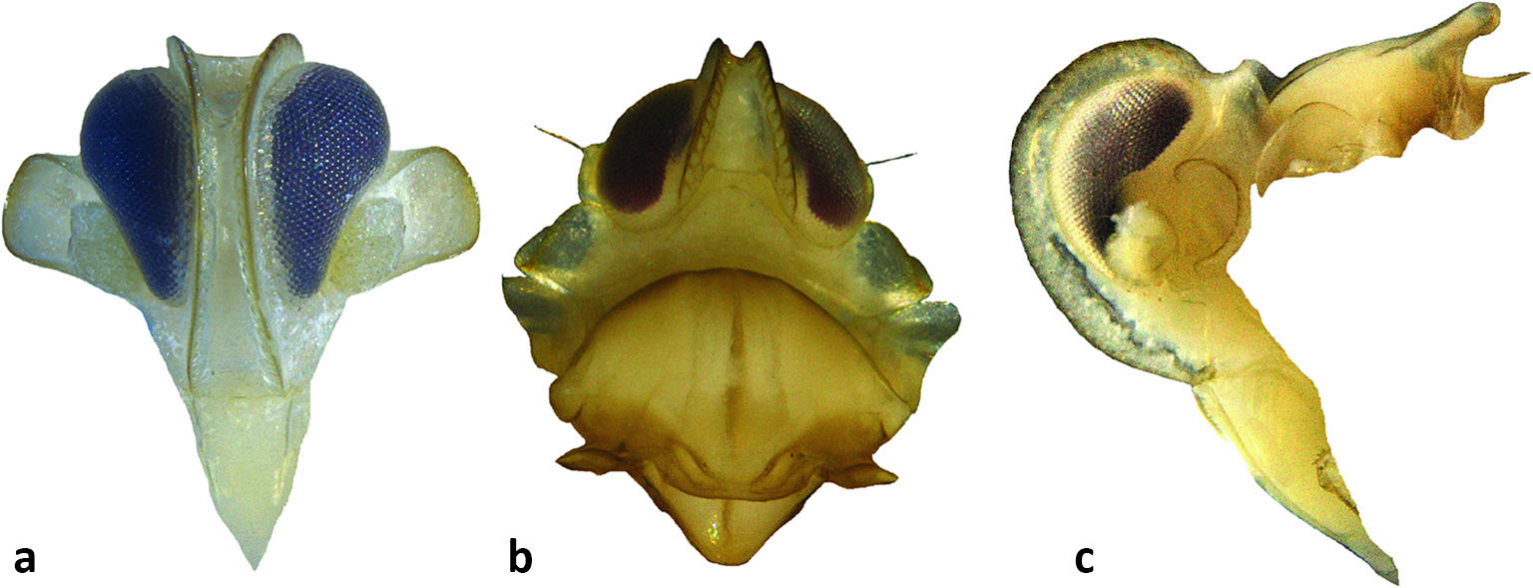
*Agoo argutiola* sp. n. (paratype), head and thorax: (A) face, frontal view; (B) head and thorax, dorsal view; (C) head and thorax, lateral view.

**Fig 6:**
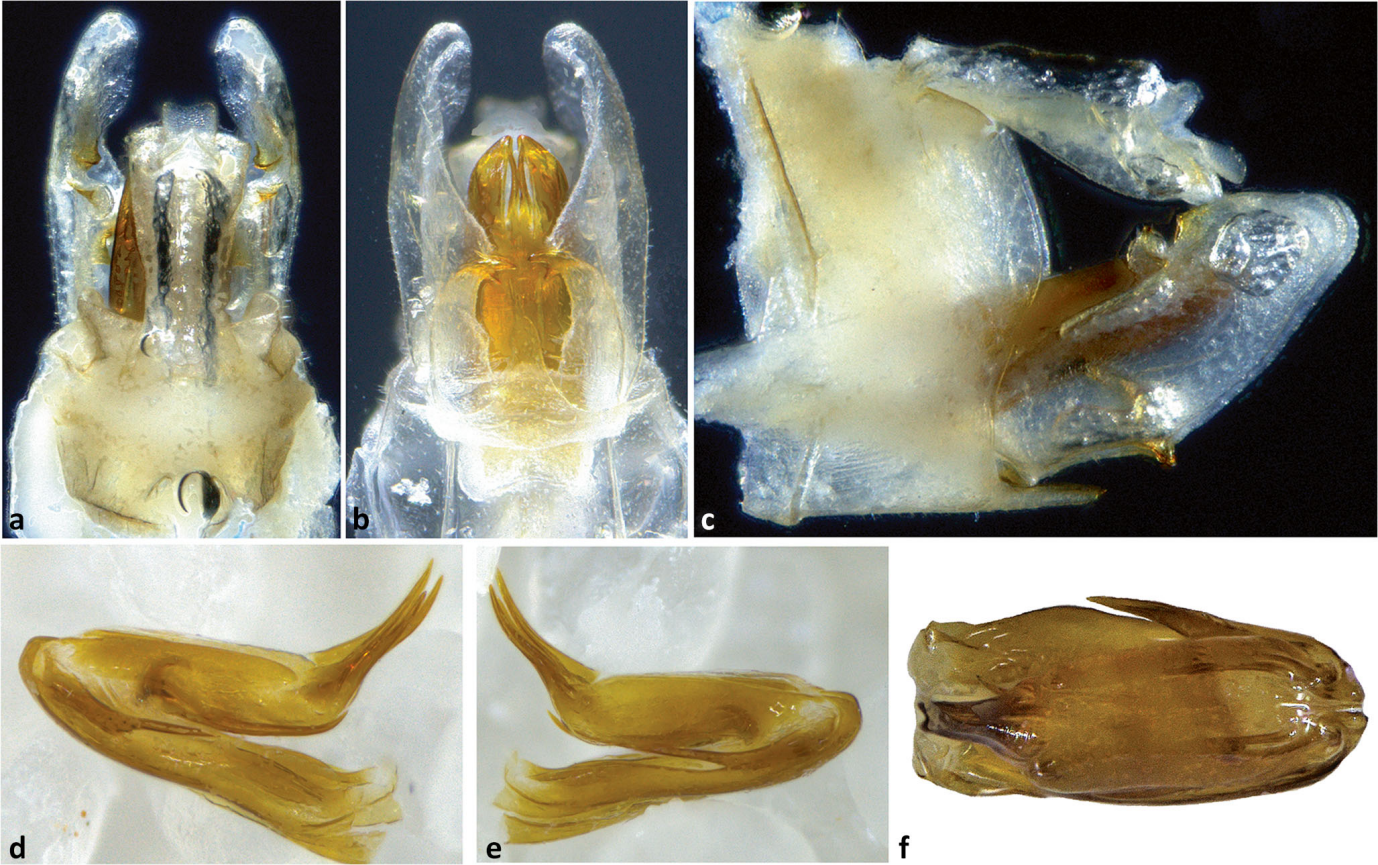
*Agoo argutiola* sp. n. (paratype): (A) terminalia, dorsal view; (B) terminalia, ventral view; (C) terminalia, lateral view; (D) extracted phallotheca, left lateral view; (E) phallotheca, right lateral view; (F) phallotheca, dorsal view (proximal portion to left).

**Fig 7:**
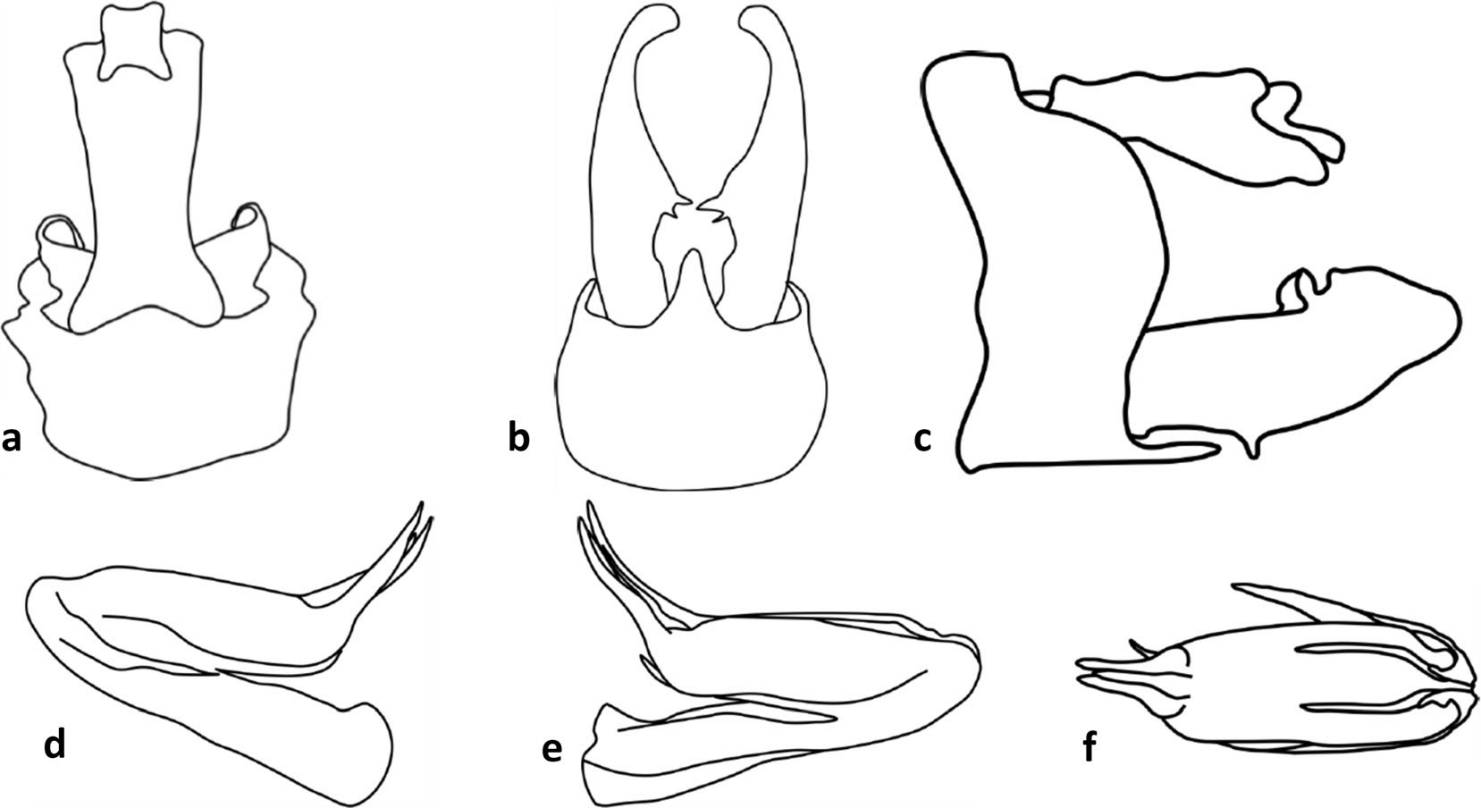
Line art for terminalia of *Agoo argutiola* sp. n. (paratype): (A) terminalia, dorsal view; (B) terminalia, ventral view; (C) terminalia, lateral view; (D) extracted phallotheca, left lateral view; (E) phallotheca, right lateral view; (F) phallotheca, dorsal view (proximal portion to left).

1. Frons and vertex relatively broad (e.g., Caldwell 1944, plate I, Fig 4), lateral margins keeled but not foliate, disk of depressed, posterior margin of vertex varies, but usually not quadrately concave; head profile in lateral view somewhat flattened on frons; phallotheca bilaterally asymmetrical, ventral lobe of pygofer opening (ventral view) varied, but often with lateroapical teeth (e.g., Caldwell 1944, Figs 5B); ventral margin of anal tube from lateral view with subapical concavity (e.g., Caldwell 1944, Fig 1C)..............................genus *Omolicna*
  – Frons and vertex narrow (Fig 5A, B), lateral margins foliate, disk deeply concave, posterior margin of vertex quadrantally concave; head in lateral view smoothly rounded from posterior margin of vertex to frontoclypeal suture (Fig 5C); phallotheca nearly bilaterally symmetrical, ventral lobe of pygofer opening (ventral view, Fig 7A) with elongately rounded lobe (subtriangular); ventral margin of anal tube sinuate, without subapical concavity (Figs 7C).................................................................. genus *Agoo ...***2**
2. Wings lacking distinct markings; Trinidad, Brazil .......... **3**
  – Wings with spots or longitudinal bands (or both); Costa Rica ............................................................................... **5**
3. Aedeagal shaft with pair of slender spines on dorsolateral margin about 2/3 from base (Fennah 1945, Fig 164); apical margin of forewing bordered with pink; Trinidad ........................................ *Agoo rubrimarginata*
  – Aedeagal shaft without lateral spines except at apex; apical margin of forewing not pink; Brazil ................... **4**
4. Lateral margin of pygofer opening with acuminate medially directed lobe (Fig 10G); gonostyli in ventral view with medially directed lobe irregularly lobed on trailing margin (Figs 10H and 11B); phallothecal flagellum with a pair of dorsal elongate retrorse processes (Fig 10D, E)......................................................... *Agoo spina* sp. n.
  – Lateral margin of pygofer opening lacking acuminate medially directed lobe (Fig 7C); gonostyli in ventral view with medially directed lobe bearing 2 short sclerotized spines on inner margin (Fig 7B); phallothecal flagellum without dorsal retrorse processes (Figs 6D, E) ....................................................... *Agoo argutiola* sp. n.
5. Wings with black spots, without distinct stripe (Bahder *et al* 2020a, Fig 7); dorsal surface of gonostyli bearing large lobe with a median invagination resulting in two processes (Bahder *et al* 2020a, Fig 10A), pair of processes on aedeagus situated posterior .................. *Agoo dahliana*
  – Wings with distinct longitudinal stripe; processes lacking on dorsal surface of gonostyli ........................................... **6**
6. Stripe on forewing terminating in red with distal black spot (Bahder *et al* 2020b, Fig 5); gonostyli with sclerotized ridge on outer lateral margin near midlength bearing sharp projection (Bahder *et al* 2020b, Fig 6A) ................................. *Agoo luzdenia*
  – Stripe on forewing fuscous (Bahder *et al* 2019, Fig 2), gonostyli lacking lateral ridge (Bahder *et al* 2019, Fig 4A) ........................................................................... *Agoo xavieri*

**Fig 8:**
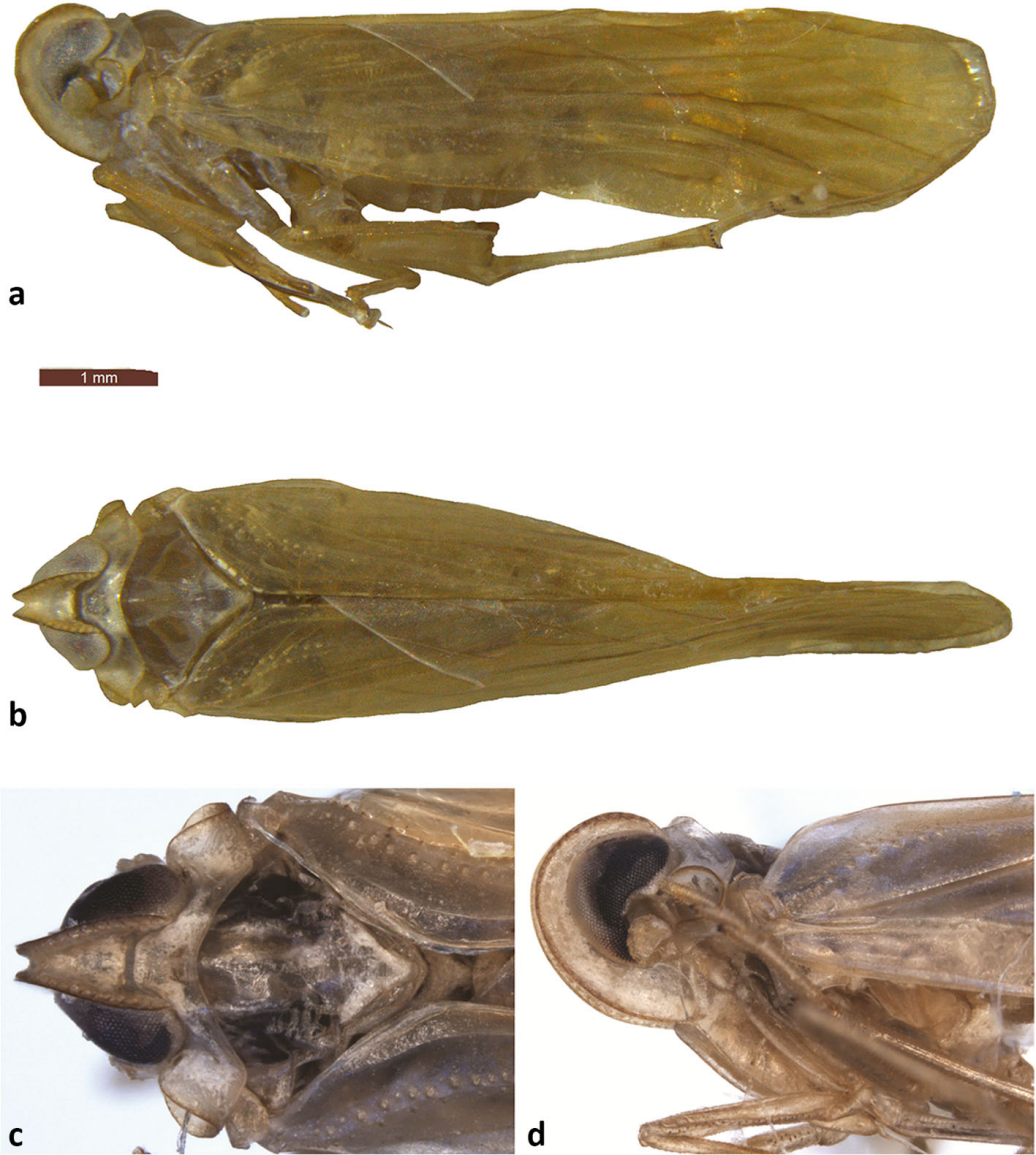
*Agoo spina* sp. n. (holotype): (A) lateral view, habitus; (B) dorsal view habitus; (C) head and thorax, dorsal view; (D) head and thorax, lateral view.

**Fig 9:**
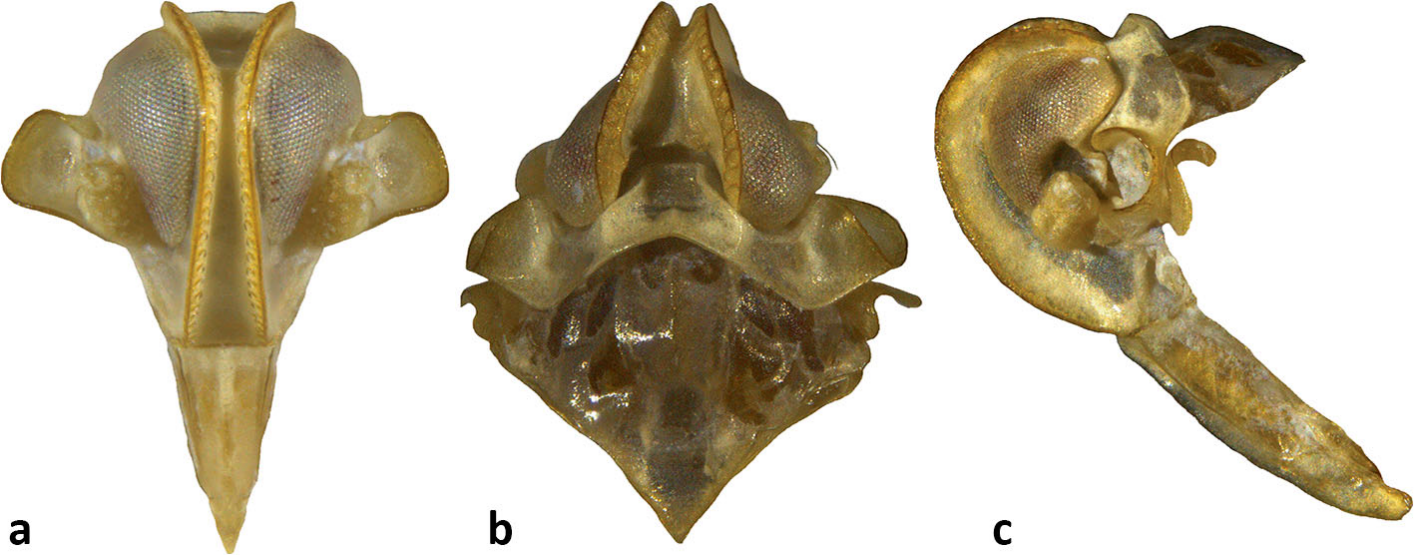
*Agoo spina* sp. n. (paratype), head and thorax: (A) face, frontal view; (B) head and thorax, dorsal view; (C) head and thorax, lateral view.

**Fig 10:**
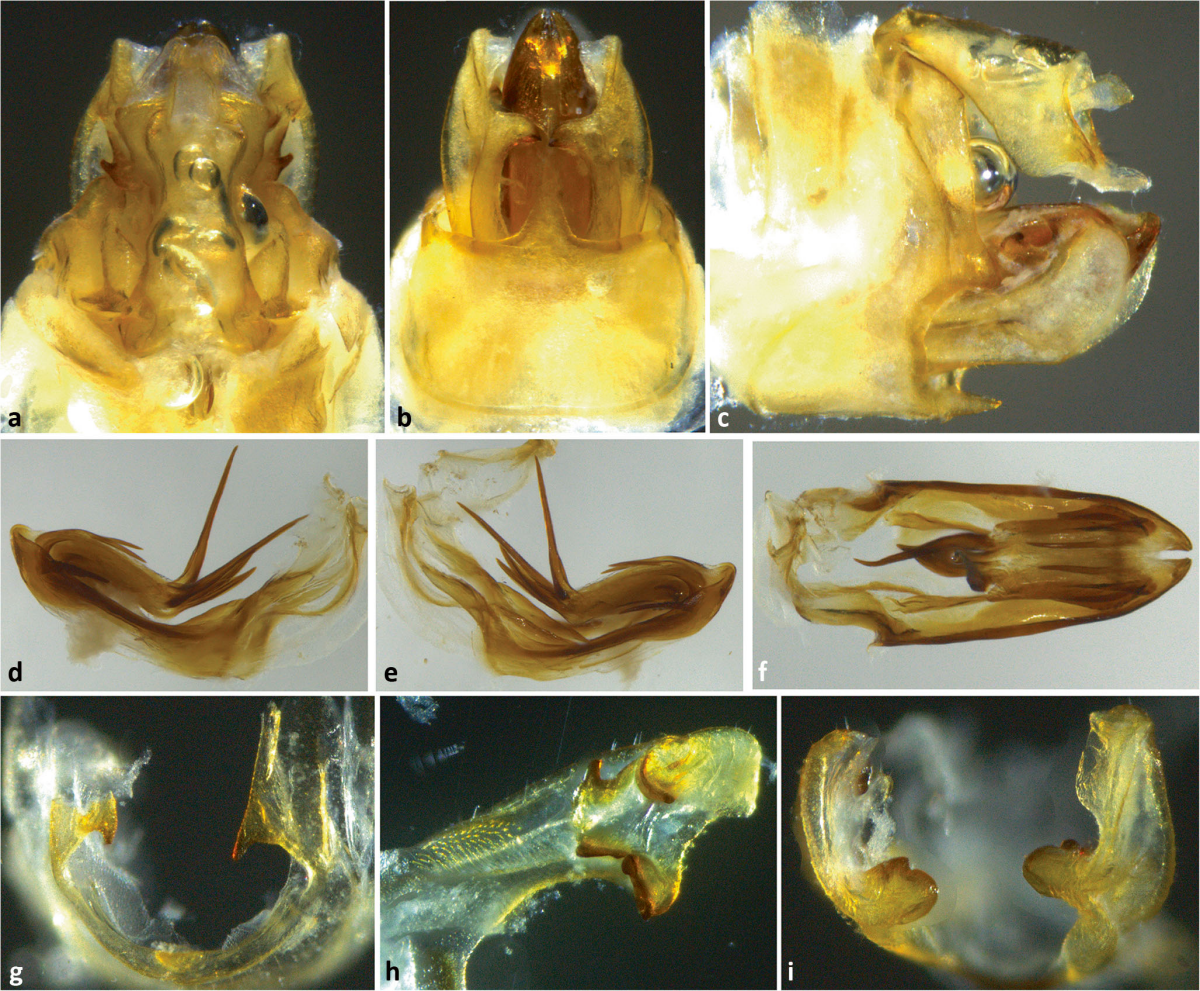
Terminalia of *Agoo spina* sp. n. (paratype): (A) Terminalia, dorsal view; (B) terminalia, ventral view; (C) terminalia, lateral view; (D) extracted phallotheca, left lateral view; (E) phallotheca, right lateral view; (F) phallotheca, dorsal view (proximal portion to left); (G) caudal view of pygofer showing projections on lateral margin of opening; (H) detail of median lobe of gonostyli showing irregularly tri-lobed trailing margin; (I) apex of gonostyli, caudal view.

**Fig 11:**
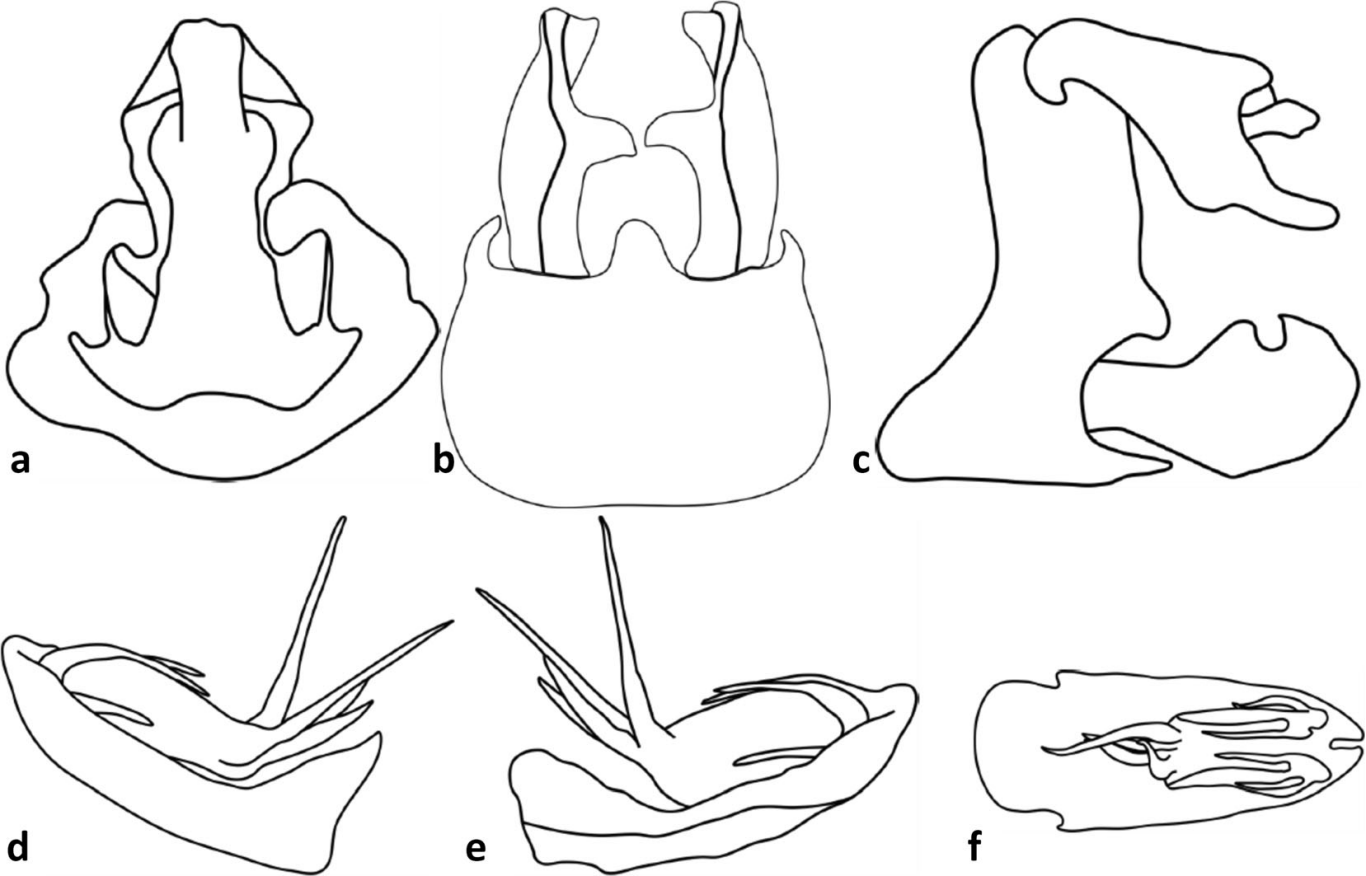
Line art of terminalia of *Agoo spina* sp. n. (paratype): (A) terminalia, dorsal view; (B) terminalia, ventral view; (C) terminalia, lateral view; (D) extracted phallotheca, left lateral view; (E) phallotheca, right lateral view; (F) phallotheca, dorsal view (proximal portion to left).

**Fig 12:**
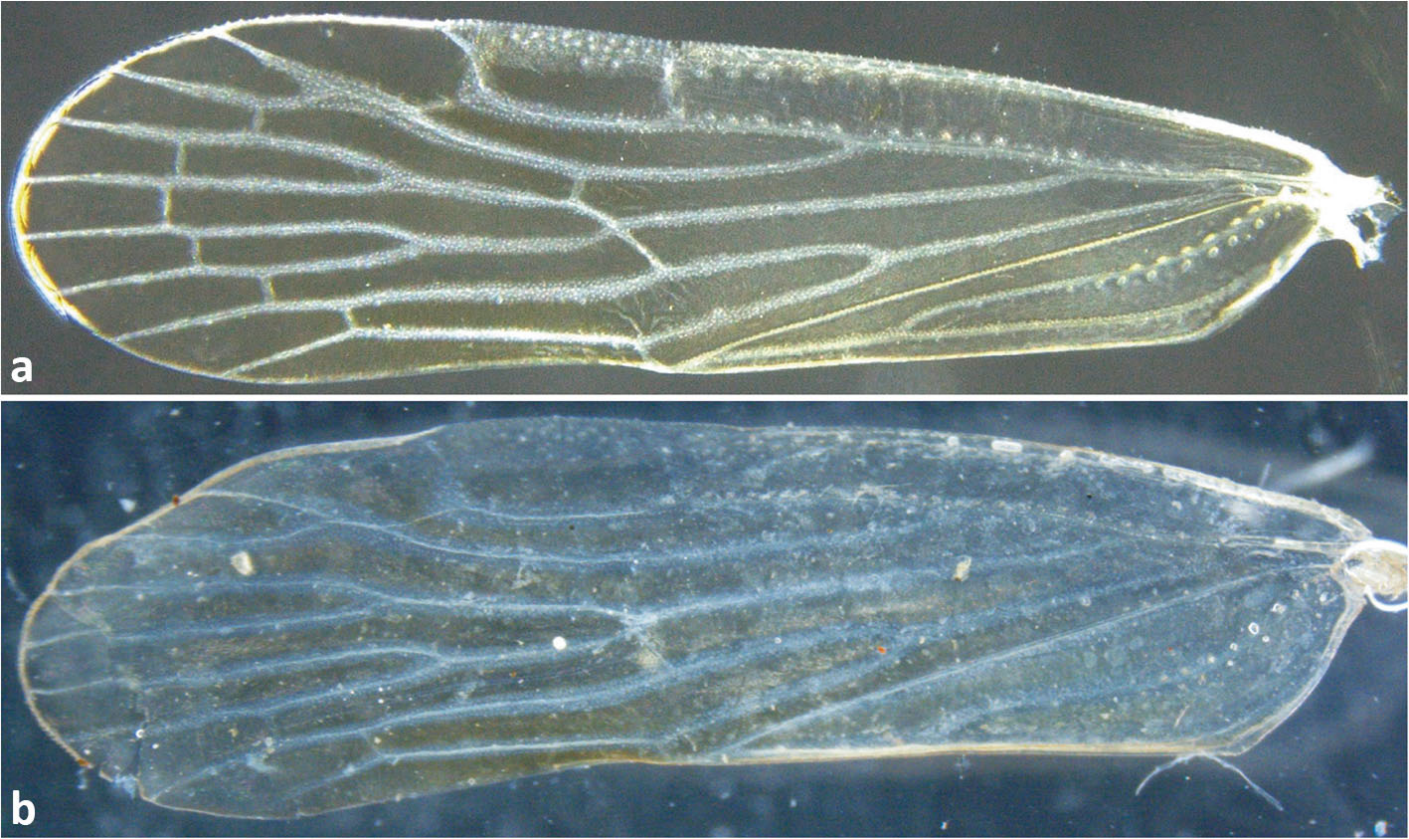
Forewings (A) *Agoo argutiola* sp. n.; (B) *Agoo spina* sp. n.

**Fig 13:**
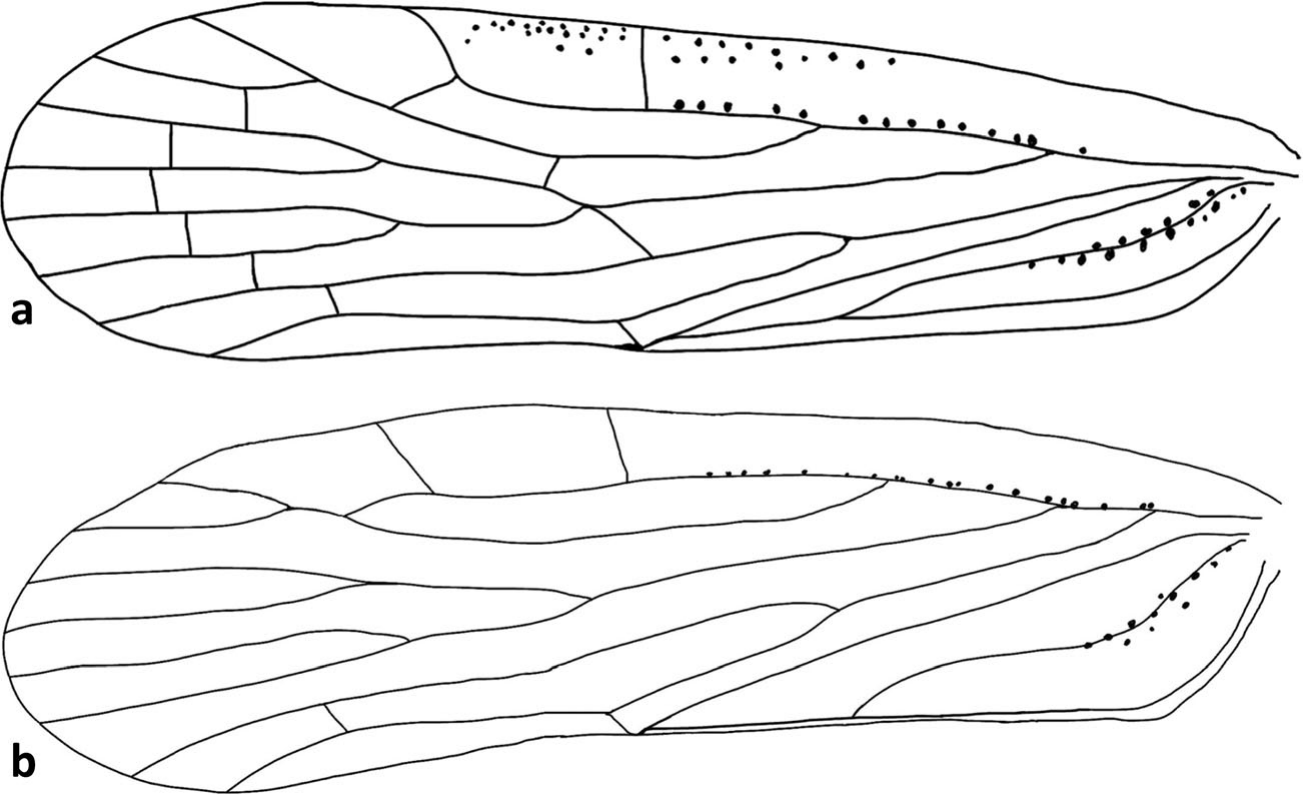
Forewing venation, line art: (A) *Agoo argutiola* sp. n.; (B) *Agoo spina* sp. n.

***Agoo argutiola*** **Bahder & Bartlett sp. n.**

(Figs 4, 5, 6, 7, 12A, and 13A)

**Type locality.** Roraima, Rorainopolis, Nova Colina.

**Diagnosis.** Male pygofer with median ventral process broad near base, attenuating distally to broadly rounded apex (lateral teeth lacking); lateral margins of pygofer opening lacking transversely projecting processes. Gonostyli in ventral view with medially directed lobe bearing two sclerotized spines on inner margin. Flagellum of aedeagus lacking dorsal processes arising near junction of aedeagal shaft.

**Description*.*** *Color*. General body color pale, stramineous; face orange on dorsal portion of frons and genae; however, some individuals are a uniform pale yellow (Fig 5A); lateral carinae of front and vertex darker. Wings transparent (Fig 12A), almost white basally, diffusely infuscated distally.

*Structure.* Body length (without wings) males: 2.99–3.45 mm (*n* = 2), females: 2.94–3.52 mm (*n* = 2); body length (with wings): not available (specimens with wings splayed). Head. Anterior margin of head, in lateral view, rounded (Fig 5C) from posterior margin of vertex to frontoclypeal suture. Vertex deeply depressed (Fig 5B), median carinae absent, quadrantally concave posteriorly, notched distally, broadest near base, tapering anteriorly; lateral carinae foliately keeled, bearing row of (sensory?) pits. Transverse apical carina at fastigium absent. Vertex length males: 0.39–0.45 mm; females: 0.41–0.43 mm. Vertex width at hind margin males: 0.33–0.41 mm; females: 0.37–0.38 mm. Vertex width at distal margin males: 0.11–0.13 mm; females: 0.12–0.14 mm. Frons with lateral carinae strongly keeled, narrowest between compound eyes, diverging slightly ventral to become parallel between ocelli and frontoclypeal suture (Fig 5A); pits on foliate carinae for entire length, median carinae absent. Lateral ocelli faintly indicated. Antennal scape very short (~ 0.09 mm), pedicel larger (~  0.21 mm), bulbous, with irregularly arranged sensillae, flagellum short (~ 0.20 mm). Frons length males: 0.84–0.86 mm; females: 0.84–1.01 mm. Frons dorsal width males: 0.10–0.11 mm; females: 0.12–0.13 mm. Frons frontoclypeal margin width, males: 0.22 mm; females: 0.22–0.26 mm. Clypeus elongate, broadest near frontoclypeal margin, tapering distally to labrum; median carina present, lateral carinae weakly keeled, pits absent. Clypeus length males: 0.62–0.68 mm; females: 0.62–0.71 mm.

Thorax. Pronotum short (length at midline males: 0.23–0.27 mm; females: 0.24–0.25 mm), anterior margin following contours of head (Fig 5B; medially convex and truncate behind vertex, narrowed behind eyes). Median carina of pronotum obsolete, lateral carinae distinct, reaching caudal margin; anterior keel following anterior margin of pronotum behind eye to antennal fossa in paradiscal field (Fig 5C); in lateral view, posterior margin of pronotum inflected upward; paradiscal field with deep fossae with foliately keeled margins (forming “cup” posterior to antennae, partially surrounding antennae both dorsad and ventrad. Mesonotum appearing slightly elevated in lateral view (Fig 5C), in dorsal view with 3 subparallel longitudinal carinae, indistinctly reaching scutellum; scutellum distinctly indicated by transverse groove, posteriorly inflected upward, dorsolateral angles of scutellum bearing projections (may be hidden by forewings in repose). Mesonotum length at midline (including scutellum) males: 0.68–0.75 mm; females: 0.50–0.65 mm. Mesonotum width males: 0.93–1.05 mm; females: 0.91–0.95 mm.

Forewing (Figs. 12A and 13A) with a row of pits along basal 1/2 of ScP+R and basal half of postcubitus. Cluster of pits between RA and ScP and two rows of four sensory pit basad of ScP. Forks of R and CuA veins at approximately the same level, both well proximad of claval apex, but near level of Pcu+1A fusion. Claval apex just near midpoint of wing, fork of M near claval apex. Branching pattern: Sc and RA unbranched, RP 2-branched, MP 4-branched and CuA 2-branched plus short branch at claval apex. Fusion of Pcu+A1 in proximal third (A1 closely approximate to trailing margin of wing). Forewing length males: 5.4–5.8 mm; females: 5.5–5.9 mm.

Terminalia. Pygofer, in lateral view, widest at base, abruptly narrowed to irregularly sinuate margins, anterior concave, caudal convex (Fig 7C). In ventral view, opening of pygofer with midventral elongately rounded lobe (Fig 7B), attenuating distally to rounded apex. Gonostyli broad and cupped (in lateral view, Fig 7C), expanded distally; dorsal margin near midlength bearing a rounded projection with a laterally directed sclerotized tooth (gonostyli distally expanded, giving the appearance of a notch after this projection). Gonostyli in ventral view (Fig 7B) with medially directed projection near midlength bearing two short sclerotized spines on inner margin, distal spine about twice as large as basal spine. Apodemes of gonostyli conspicuous, extending beyond pygofer into abdomen. Phallotheca stout (Fig 6D–F), approximately bilaterally symmetrical; shaft of phallotheca with elongate retrorse processes near apex (each with irregularly arranged serrations near midlength), one each side of large retrorse flagellum; flagellum with 2 pairs of elongate apical processes (apparently articulated, about equal-sized) arranged in transverse row (Figs 6D, E); and 1 elongate midventral process. Anal tube in lateral view roughly triangular, distally broadened, ventrally sinuate, caudal margin concave at anal column, ventrocaudal portion elongate (apically rounded) (Fig 7C). In dorsal view, anal tube notched on caudal aspect; anal column short, appearing quadrate.

**Plant associations.** Oil palm (*Elaeis guineensis* Jacq.), and coconut (*Cocos nucifera* L), both Arecaceae.

**Distribution.** Brazil (Roraima).

**Etymology.** The specific name *argutiola* is a Latin diminutive of *argutia*, meaning a piece of subtlety, a reference to some of the diagnostic features in *Omolicna*. The name is feminine in gender.

**Material examined.** HOLOTYPE male “22933 S 28 / (cig. Branca) / Bresil: Roraima / Nova Colina // 3.VIII.2016 / *Elaeis guineensis* / Dollet & Fidelis *Leg.* // UDCC_TCN 00058471 [2d barcode]// HOLOTYPE / *Agoo argutiola* / Bahder & Bartlett” (1 male, dissected, MPEG). PARATYPES, same data (2d barcode numbers: UDCC_TCN 00097147, UDCC_TCN 00097148) (2 males, MPEG); same data, UDCC_TCN 00097146, 00°36′48.7″N, 60°17′39.7″W, in alcohol, 2 females, UDCC); Roraima, Boa Vista, 2°47′02.6″N, 60°45′12.5″W, coconut, 27–VII–2016, Michel Dollet & Elisangela Fidelis leg (1 male, UDCC); Roraima, São João da Baliza, 0°56′07.9″N 59°52′40.4″W, coconut, 21–III–2017, Michel Dollet & Elisangela Fidelis leg. (sample SJ4b, 1 male, UDCC); same, dated (20–21)–III–2017, coconut (an additional specimen from this locality appears to have different terminalia and is excluded from the paratype series).

***Agoo spina*** **Bahder & Bartlett sp. n.**

(Figs 8, 9, 10, 11, 12B, and 13B)

**Type locality.** Brazil, Sergipe, Itaporanga D’Ajuda.

**Diagnosis.** Male pygofer with median ventral process broad near base, attenuating distally to broadly rounded apex (lateral teeth lacking); lateral margins of pygofer opening with acuminate transversely projecting processes. Gonostyli in ventral view with acuminate medially directed lobe, trailing edge of lobe irregularly tri-lobed. Flagellum of aedeagus with pair of dorsal retrorse processes arising near junction with aedeagal shaft.

**Description*.*** *Color*. General body color is uniformly tan (Fig 8). Carinae on face darker. Mesonotum darker on lateral portions (pale medially). Forewings transparent (Fig 12B), veins pale; weakly infuscated with dark in basal third (except leading margin), faintly infuscated more distal.

*Structure.* Body length males (with wings): 6.89–6.95 mm (*n* = 2) with wings; females not available for study; body length (without wings): not available (dissected). Head: Anterior margin of head, in lateral view, smoothly rounded from posterior margin of vertex to frontoclypeal suture (Figs 8A, D and 9C). In dorsal view, vertex quadrately concave posteriorly between compound eyes, medially truncate (Fig 9B). Vertex disc medially deeply depressed between foliately carinate lateral margins (median carina absent); lateral margins most widely separated posteriorly, converging anteriorly to narrow fastigium; lateral keels of vertex bearing a row of (sensory?) pits. Transverse carina of fastigium absent. Vertex length (males): 0.31–0.32 mm; width (hind margin) 0.33–0.34 mm; vertex width (apical margin) 0.12–0.14 mm. Frons elongate, medially depressed (Fig 9A, median carina absent), lateral keels strongly foliate, bearing row of pits, continuous with vertex. Frons narrowest between compound eyes, broadening to frontoclypeal suture. Frons length (males): 0.85–0.87 mm; frons width (narrowest, between eyes): 0.12–0.14; width (frontoclypeal margin): 0.33–0.34 mm. Ocelli obsolete. Antennae stout, scape very short (~ 0.05 mm), pedicle rather rounded (almost mushroom-shaped, ~ 0.25 mm), bearing irregularly arranged sensillae, flagellum not evident (possibly broken). Clypeus elongate, broadest at frontoclypeal martin, narrowing distally to labrum; median carinae present, lateral carinae weakly carinae keeled, pits wanting. Clypeus length: 0.61–0.63 mm.

Thorax. Pronotum narrow (length at midline 0.21–0.25 mm), anterior margin following contours of posterior margin of head (Fig 9B); medially convex and truncate behind vertex, narrowed behind eyes. Posterior pronotal margin medially concave; median carina of pronotum very weak, lateral carinae distinct, reaching caudal margin; anterior keel following anterior margin of pronotum behind eye to antennal fossa in paradiscal field. In lateral view (Fig 9C), posterior margin of pronotum foliate, appearing inflected upward; lateral portion of paradiscal field forming strongly foliate fossa, enveloping posterior margin of antennae, and partially surrounding antennae both dorsally and ventrally. Mesonotum appearing slightly elevated in lateral view; in dorsal view with median and 2 lateral longitudinal carinae, reaching scutellum. Scutellum separated from scutum by inflection, dorsolateral angles of scutellum bearing lateral projections (may be hidden by forewings in repose). Mesonotum length at midline 0.68–0.77 mm, width at tegulae 0.75–0.81 mm.

Forewing (Figs 12B and 13B) with a row of pits along basal 1/2 of ScP+R (+M) and basal half of postcubitus. Venation ScP+RA forked from RP in basal 1/3 of wing. Fork of CuA and fusion of Pcu+1A at nearly same level; ScP (unbranched) from RA at about wing midlength; RP 2-branched; MP 4-branched; CuA 2-branched plus short branch near apex of clavus; claval apex at nearly wing midlength; A1 tracking wing margin (wing margin inflected to be obscured in lateral view of body); combined Pcu+1A reaching margin before apex of clavus.

Terminalia. Pygofer in lateral view widest near base (Fig 11C), proximal and distal margins irregularly sinuate, subparallel; proximal margin concave, distal convex with an acuminate transversely projecting process just below midlength (Fig 10G). Pygofer opening in ventral view bearing an elongate lobe, widest at base and attenuating distally to rounded apex. Gonostyli (in lateral view) broad (Fig 11C), distally expanded into rounded, cup-like structure; apex bluntly rounded; dorsal subapical region with pair of asymmetrical lobes, each bearing a large, the distal lobe with the tooth directed medially, the proximal lobe with tooth directed laterad; in ventral view (Fig 11B), each gonostylus bearing a large, triangular lobe directed dorsomedially, their dorsal surfaces sclerotized and irregularly lobed (Fig 10H). Apodemes from gonostyli elongate and conspicuous projecting into abdomen beyond pygofer. Phallotheca stout (Fig 10D–F), nearly bilaterally symmetrical; shaft with small laterodorsal subbasal tooth on each side and elongate retrorse process arising at each side of flagellum base (these appearing partly serrulate in dorsal view); apex of shaft subtended by pair of cupped projections from ventral surface (giving appearance of apical notch in dorsal view); aedeagal flagellum strongly retrorse bearing 4 pairs of elongate processes, 1 dorsal pair near flagellar base, 1 pair on flagellar underside, arising near base and 2 apical pairs arranged in a roughly transverse row (lateral pair longer), appearing articulated at base (Fig 10D, E). Anal tube stout in lateral view broadly triangular, broadened caudally, dorsal margin straight to anal column, ventral margin sinuate, apex rounded; in dorsal view caudal margin apically concave anal column short (Fig 11C).

**Plant associations.** Coconut palm (*Cocos nucifera*), Arecaceae.

**Distribution.** Brazil (Sergipe).

**Etymology.** The species name is from the Latin term *spina* meaning thorn, a reference to the process on the lateral margins of the pygofer opening. The term is intended to be feminine in gender.

**Material examined.** HOLOTYPE male “Brazil, Sergipe / Itaporanga D’Ajuda / 22-Jul-17 / Coconut, sweeping net / Eliana Passos // UDCC_TCN 00097417 [2d barcode label]// *Agoo argutiola* / Bartlett & Bahder” (dissected male, MPEG). PARATYPES: BRAZIL, Sergipe, Itaporanga D’Ajuda, Active germplasm bank of coconut; 19.V.2016; aspirated from Coconut, Eliana Passos, UDCC_TCN 00097419 (2d barcode label) (2 males, dissected, UDCC).

**Remarks.** *Agoo spina* sp. n. and *Agoo argutiola* sp. n. both differ from *Agoo xavieri* in the form of the flagellum of the phallotheca—both of the new species possess a terminal transverse row of 4 processes, whereas *A. xavieri* does not. *Agoo spina* and *Agoo argutiola* are quite similar except that the former possesses an acuminate medially directed lobe on the lateral margin of pygofer opening (lacking in *A. argutiola*) and possesses a pair of elongate retrorse processes on the dorsum of the flagellum (lacking in *A. argutiola*).

## Discussion

In this work, we document eight species of planthoppers in the family Derbidae associated with coconut or oil palm in Brazil. Undoubtedly, there are other derbid species to be found associated with palms. Additional derbids not reported here were collected in Bonfim (East of Boa Vista), Boa Vista, Nova Colina, and Mucajai that remain to be investigated, including specimens similar to *Omolicna* and *Cedusa*. The presence and abundance of derbids on palms has been observed many times; for example, Howard (2001) lists 91 derbid species reported from palms, nearly doubling the number compiled by Lepesme (1947). Zelazny & Pacumbaba (1982) report collecting 4978 derbid specimens representing 15 taxa from coconut palms in Luzon, Philippines. Wilson *et al* (1994) report 36% of the derbid plant association records compiled by them are from palms, and 63% of derbid species with host associations are reported from a single plant species. Available data in FLOW (Bourgoin 2020) indicate that 16.7% of published plant associations for Derbidae are Arecaceae, more than any other plant family (next are Poaceae and Sapindeceae at 11.4% each).

The nymphal stages of derbids are associated with moist organic debris (e.g., Wilson 1987a, Wilson *et al* 1994, Howard *et al* 2001) and are assumed to feed on fungal hyphae (e.g., Wilson *et al* 1994, Bartlett *et al* 2014), and adults move to plants to feed and mate. It is possible that the association between palms and derbids (at least for some derbid species) has less to do with nutrition, and more to do with relationships between palms and derbid larval habitat, or plant structure or allelochemistry as they relate to mate-finding or substrate-born communication (Wilson 1987b).

While the association between some derbids and palms is evident from current survey efforts in Costa Rica (Bahder *et al* 2019, 2020a, b), the biological significance is not. Habitats where members of the genus *Agoo* have been sampled extensively and in great numbers have failed to identify non-palm host plants (Bahder, *unpublished data*). There has been no unequivocal evidence that derbids transmit palm pathogens, which remains a critical issue. Powell *et al* (2015) documented the presence of the 16SrIV-D phytoplasma in the newly discovered *Omolicna joi*; however, this was based on DNA extraction from the whole body of the insect. Detection of phytoplasma from whole-body extractions of insects has some use in vector studies because it allows for the identification of species that are in fact feeding on infected palms. However, positive reactions using this approach are highly susceptible to obtaining false positives. Screening for phytoplasmas is commonly done using the 16S gene which is highly conserved and commonly results in the amplification of other species of bacteria from the insect gut or that it has acquired through feeding (Wally *et al* 2008). Another form of false positive is when the whole body is tested and true phytoplasma DNA is amplified but is residual phytoplasma passing through the gut. Residual pathogens can be detected in non-vectors (Cieniewicz *et al* 2018) so to understand if derbids do contribute to transmission of phytoplasmas, screening of salivary glands will need to be performed on specimens to assess if they are capable of transmitting the phytoplasma in question.

That two of the species we found had not been previously described is a testament both to the incompleteness of past survey work of palm-associated planthoppers, and the taxonomic challenges associated with the derbid tribe Cenchreini. Among the Cenchreini, there has been no quantitative phylogenetic treatments at any level and the diagnostic morphological differences among genera are incompletely defined. Diagnostic molecular work to supplement species descriptions and test generic placement (e.g., to confirm placement of the new species described here in the genus *Agoo*) and monophyly are needed both to amend basic science (i.e., taxonomy, especially to define species, investigate intraspecific variation and confirm associations of males, females and nymphs) but also to provide additional diagnostic and applied tools.

### Nomenclature

ZooBank registration can be found at: urn:lsid:zoobank.org:pub:B6D0575E-1C39-4825-8DA5-0879A1FF6BB5; *Agoo argutiola* Bahder and Bartlett urn:lsid:zoobank.org:act:CA623FE3-A7CA-432A-83B4-DE3145CEBEF2; *Agoo spina* Bahder and Bartlett urn:lsid:zoobank.org:act:194C7BDB-B2D8-446B-B96C-C913553C25D7.

## References

[CR1] Arthropod Easy Capture (2013) An arthropod specific, specimen level data capture application, Version: 1.34. https://sourceforge.net/projects/arthropodeasy. Accessed 3 July 2019

[CR2] Bahder BW, Bartlett CR, Barrantes EA, Echavarria MAZ, Humphries AR, Helmick EE, Ascunce MS, Goss EM (2019). A new species of *Omolicna* (Hemiptera: Auchenorrhyncha: Fulgoroidea: Derbidae) from coconut palm in Costa Rica and new country records for *Omolicna brunnea* and *Omolicna triata*. Zootaxa.

[CR3] Bahder BW, Bartlett CR, Helmick EE, Barrantes EA, Echavarria MAZ, Goss EM, Ascunce MS (2020). Revised status of *Omolicna* subgenus *Agoo* (Hemiptera: Auchenorrhyncha: Fulgoroidea: Derbidae) with a new species from Costa Rica and new country records. Zootaxa.

[CR4] Bahder BW, Zumbado Echavarria MA, Barrantes EA, Bartlett CR, Helmick EE, Kunz G, Ascunce MS (2020b) A new species of planthopper in the genus *Agoo* (Hemiptera: Fulgoroidea: Derbidae) from coquito palms (*Astrocaryum alatum*) in Costa Rica. Zootaxa (in press) 10.11646/zootaxa.4779.3.8 33055782

[CR5] Balick MJ (1979). Amazonian oil palms of promise: a survey. Econ Bot.

[CR6] Bartlett CR, O’Brien LB, Wilson SW (2014). A review of the planthoppers (Hemiptera: Fulgoroidea) of the United States. Mem Am Entomol Soc.

[CR7] Bourgoin T, Vidano C, Arzone A (1988). A new interpretation of the homologies of the Hemiptera male genitalia illustrated by the Tettigometridae (Hemiptera, Fulgoromorpha). Proceedings of the 6th Auchenorrhyncha meeting.

[CR8] Bourgoin T (2020) FLOW (Fulgoromorpha lists on the web): a world knowledge base dedicated to Fulgoromorpha. Version 8 (updated 24 April 2020). http://hemiptera-databases.org/flow/ Accessed 30 April 2020

[CR9] Bourgoin T, Huang J (1990). Morphologie comparée des genitalia mȃles des Trypetimorphini et remarques phylogénétiques (Hemiptera: Fulgoromorpha: Tropiduchidae). Ann Soc Entomol Fr (Nouvelle Serie).

[CR10] Bourgoin T, Wang RR, Asche M, Hoch H, Soulier-Perkins A, Stroinski A, Yap S, Szwedo J (2015). From micropterism to hyperpterism: recognition strategy and standardized homology-driven terminology of the forewing venation patterns in planthoppers (Hemiptera: Fulgoromorpha). Zoomorphology.

[CR11] Brondizio ES, Pinedo-Vasquez M, Ruffino ML, Padoch C, Brondízio ES (2011). Forest resources, family networks and the municipal disconnect: examining recurrent underdevelopment in the Amazon estuary. The Amazon Várzea: the decade past and the decade ahead.

[CR12] Brown SE, Been BO, McLaughlin WA (2006). Detection and variability of the lethal yellowing group (16Sr IV) phytoplasmas in the *Cedusa* sp. (Hemiptera: Auchenorrhyncha: Derbidae) in Jamaica. Ann Appl Biol.

[CR13] Caldwell JS (1944). The tribe Cenchreini with special references to the *Cenchrea* complex (Homoptera: Derbidae). Bull Brooklyn Entomol Soc.

[CR14] Cieniewicz EJ, Pethybridge SJ, Loeb G, Perry KK, Fuchs M (2018). Insights into the ecology of *Grapevine red blotch virus* in a diseased vineyard. Phytopathology.

[CR15] Clair D, Larrue J, Boudon-Padieu E (2001). Evaluation of vectoring ability of phytoplasmas by *Metcalfa pruinosa* say (Homoptera: Flatidae) recently introduced in Europe. International Organization for Biological and Integrated Control of Noxious Animals and Plants, West Palearctic Regional Section (IOBC wprs). Bulletin.

[CR16] Dollet M, Talamani V, Fidelis E, Lohmann TR, Silva ML, Parizzi P, Laranjeira FF (2018). Fitoplasmas associados às Síndromes do Tipo Amarelecimento Letal das Palmeiras. Priorização de pragas quarentenárias ausentes no Brasil.

[CR17] Dollet M, Quaicoe R, Pilet F (2009). Review of coconut “lethal yellowing” type diseases diversity, variability and diagnosis. OCL.

[CR18] Eden-Green SJ, Eden-Green SJ, Ofori F (1997). History and world distribution of lethal yellowing-like diseases of palms.

[CR19] Emeljanov AF (1992). Two new tribes, a new genus and a new species of the family Derbidae (Homoptera: Fulgoroidea). Vestnik Zool.

[CR20] Emeljanov AF (1996). On the system and phylogeny of the family Derbidae (Homoptera, Cicadina). Entomol Rev.

[CR21] Emeljanov AF (2008). New species of the genus *Peltonotellus* Puton (Homoptera, Caliscelidae) from Kazakhstan, Middle and Central Asia. Tethys Entomol Res.

[CR22] FAO (2020) FAOSTAT food and agriculture data. http://www.fao.org/faostat/en/#home/. Accessed 03 Mar 2020

[CR23] Fennah RG (1945). The Fulgoroidea, or lanternflies, of Trinidad and adjacent parts of South America. Proc U S Natl Mus.

[CR24] Fennah RG (1952). On the generic classification of Derbidae (Fulgoroidea), with descriptions of new neotropical species. Trans R Entomol Soc Lond.

[CR25] Fennah RG (1961). Le Parc National de Niokolo-Koba. XXXIII Homoptera Fulgoroidea. Mém l’Instiut Fr l’Afr Noire.

[CR26] Flynn JE, Kramer JP (1983). Taxonomic study of the planthopper genus *Cedusa* in the Americas (Homoptera: Fulgoroidea). Entomography.

[CR27] Fontes HR, Wanderley M (2006) Situação atual e perspectivas para a cultura do coqueiro no Brasil. Embrapa Tabuleiros Costeiros, Aracaju, p 16 (Documentos, 94) http://www.cpatc.embrapa.br/publicacoes_2006/doc-94.pdf/. Accessed 03 Feb 2020

[CR28] Halbert SE, Wilson SW, Bextine B, Youngblood SB (2014). Potential planthopper vectors of palm phytoplasmas in Florida with a description of a new species of the genus *Omolicna* (Hemiptera: Fulgoroidea). Fla Entomol.

[CR29] Howard FW, Howard FW, Moore D, Giblin-Davis RM, Abad RG (2001). Sap-feeders on palms.

[CR30] Howard FW, Weissling TJ, O'Brien LB (2001). The larval habitat of *Cedusa inflata* (Hemiptera: Auchenorrhyncha: Derbidae) and its relationship with adult distribution on palms Florida. Entomologist.

[CR31] IBGE (2018) Censo Agropecuário 2017. https://sidra.ibge.gov.br/pesquisa/censoagropecuario/censoagropecuario-2017/. Accessed 06 Feb 2020

[CR32] Kahn F (1991). Palms as key swamp forest resources in Amazonia. Forest Ecol Manage.

[CR33] Kramer JP (1986). Supplement to a taxonomic study of the planthopper genus *Cedusa* in the Americas (Homoptera: Fulgoroidea: Derbidae). Entomography.

[CR34] Lee HS, Wilson SW (2010). First report of the Nearctic flatid planthopper *Metcalfa pruinosa* (Say) in the Republic of Korea (Hemiptera: Fulgoroidea). Entomol News.

[CR35] Lepesme P (1947). Les insectes des Palmiers.

[CR36] Martins CR, Jesus Júnior LA (2014) Produção e Comercialização de Coco no Brasil Frente ao Comércio Internacional: Panorama 2014. Embrapa Tabuleiros Costeiros, Aracaju, p 51 (Documentos 184) https://ainfo.cnptia.embrapa.br/digital/bitstream/item/122994/1/Producao-ecomercializacao-Doc-184.pdf/. Accessed 03 Feb 2020

[CR37] McCoy RE, Howard FW, Tsai JH, Donselman HM, Thomas DL, Basham HG, Atilano RA, Eskafi FM, Britt LL, Collins ME (1983) Lethal yellowing of palms. Agricultural Experiment Stations Bulletin 834. University of Florida, Gainesville

[CR38] McGrath P (2002) Red alert to lethal yellow. New agriculturist On line. http://www.new-ag.info/02-3/develop/dev05.html

[CR39] Metcalf ZP (1938). The Fulgorina of Barro Colorado and other parts of Panama. Bull Museum Comp Zool Harv Coll.

[CR40] Metcalf ZP (1945). Fulgoroidea (Homoptera) of Kartabo, Bartica District, British Guiana. Zoologica Scientific Contributions of the New York Zoological Society.

[CR41] Mpunami A, Tymon A, Jones P, Dickinson MJ (2000). Identification of potential vectors of the coconut lethal disease phytoplasma. Plant Pathol.

[CR42] Mtiiz-Miret N, Vamos R, Hiraoka M, Montagnini F, Mendelsohn R (1996). The economic value of managing the açai palm (*Euterpe oleracea* Mart.) in the floodplains of the Amazon estuary, Pará, Brazil. Forest Ecol and Manage.

[CR43] Muir FAG (1913). On some new species of leafhoppers Part II Derbidae. Bull Hawaiian Sugar Plant Assoc Exp Stat Div Entomol.

[CR44] Muir FAG (1918) Notes on the Derbidae in the British Museum collection-II Derbidae. Entomol Mon Mag 54:228–243

[CR45] O’Brien LB (1982). Two Neotropical derbid genera with observations on wing rolling (Fulgoroidea: Homoptera). Fla Entomol.

[CR46] O'Brien LB (1987). Corrections and additions to Metcalf’s “the Fulgorina of Barro Colorado and other parts of Panama” (Homoptera: Fulgoroidea). Ann Entomol Soc Am.

[CR47] Philippe R, Pokou Nkansah J, Fabre S, Quaicoe R, Pilet F, Dollet M (2007). Search for the vector of Cape Saint Paul Wilt (coconut lethal yellowing) in Ghana. Bull Insectol.

[CR48] Philippe R, Reignard S, Descamps S, Nkansah-Poku J, Pilet F, Fabre S, Dollet M (2009). Study on the transmission of coconut lethal yellowing in Ghana. OCL.

[CR49] Powell CM, Hail D, Potocnjak J, Hanson JD, Halbert SH, Bextine BR (2015). Bacterial community composition of three candidate insect vectors of palm phytoplasma (Texas Phoenix Palm Decline and lethal yellowing). Curr Microbiol.

[CR50] Rajan P (2013). Transmission of coconut root (wilt) disease through planthopper, *Proutista moesta* Westwood (Homoptera: Derbidae). Pest Manag Hort Ecosyst.

[CR51] Rocca MM (2013). Palm diseases in central America. Phytopathol.

[CR52] Rodrigues JVC, Segarra AE, Ramirez A (2010) Nested-PCR assays detect phytoplasma in *Cedusa caribbensis* Caldwell & Martorell (Hemiptera: Auchennorhyncha: Derbidae) in Puerto Rico. In: Proceedings of the Potential Invasive Pests Workshop, Miami, Florida, pp 59

[CR53] Schuh RT (2012). Integrating specimen databases and revisionary systematics. ZooKeys.

[CR54] Schuh RT, Hewson-Smith S, Ascher JS (2010). Specimen databases: a case study in entomology using web-based software. Am Entomol.

[CR55] Spinola M (1839). Essai sur les Fulgorelles, sous-tribu de la tribu des Cicadaires, ordre des Rhyngotes. Ann Soc Entomol Fr.

[CR56] Sullivan M, Harrison N (2013) CPHST Pest Datasheet for ‘*Candidatus* Phytoplasma palmae’ and related strains. USDA-APHIS-PPQ-CPHST. Revised June, 2014. http://download.ceris.purdue.edu/file/1910

[CR57] Szwedo J (2006). First fossil record of Cedusini in the Eocene Baltic amber with notes on the tribe (Hemiptera: Fulgoromorpha: Derbidae). Russian Entomol J.

[CR58] Tunçer B, Schroeder P (2017) Sambazon: creating environmental and social values through marketing the açai berry. In: Ursula Tischner, Stø E, Kjærnes U, Tukker A (eds) Sustainable agro-forestry practices in the Brazilian Amazon. System innovation for sustainability 3: case studies in sustainable consumption and production. Food and Agriculture. Routledge, London, pp 160-175

[CR59] Wally O, El Hadrami A, Khadhair AH, Adam LR, Shinners-Carnelley T, Elliott B, Daayf F (2008). DNA sequencing reveals false positives during the detection of aster yellows phytoplasmas in leafhoppers. Sci Hortic.

[CR60] Weintraub PG, Beanland L (2006). Insect vectors of phytoplasmas. Annu Rev Entomol.

[CR61] Wilson MR (1987). African Derbidae (Homoptera, Fulgoroidea): taxonomic notes with descriptions of new species collected mainly from coconut. J Nat Hist.

[CR62] Wilson MR, Wilson MR, Nault LR (1987). The Auchenorrhyncha (Homoptera) associated with palms. Proceedings of the 2nd International Workshop on Leafhoppers and Planthoppers of Economic Importance.

[CR63] Wilson SW (2005). Keys to the families of Fulgoromorpha with emphasis on planthoppers of potential economic importance in the southeastern United States (Hemiptera: Auchenorrhyncha). Fla Entomol.

[CR64] Wilson SW, Mitter C, Denno RF, Wilson MR, Denno RF, Perfect TJ (1994). Evolutionary patterns of host plant use by delphacid planthoppers and their relatives. Planthoppers: their ecology and management.

[CR65] Zelazny B, Pacumbaba E (1982). Phytophagous insects associated with cadang-cadang infected and healthy coconut palms in South-Eastern Luzon, Philippines. Ecol Entomol.

